# Supplementary figures and images for: Measuring spectrally-resolved information transfer
Source: PLoS Comput Biol. 2020 Dec 28;16(12):e1008526. doi: 10.1371/journal.pcbi.1008526 (PMC7793276; doi:10.1371/journal.pcbi.1008526)

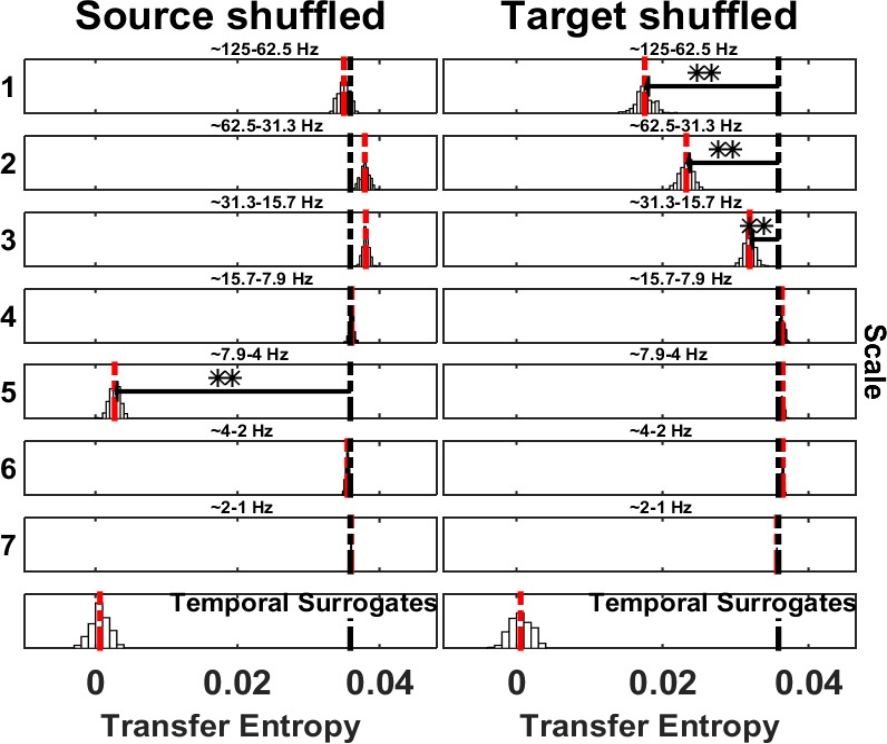

Supplement: S1 Fig — See Fig 4 for display conventions. (Left column) Information transfer, drops when wavelet coefficients are selectively shuffled at scale 5 (frequency band 4-8 Hz) on the source site. The corresponding reception of information at the target is shown on the right column, where a drop for shuffled wavelet coefficients is observed at scale 1 (frequency band 63-125 Hz), scale 2 (frequency band 31-63 Hz) and scale 3 (frequency band 16-31 Hz). (TIFF) [file pcbi.1008526.s001.tiff]

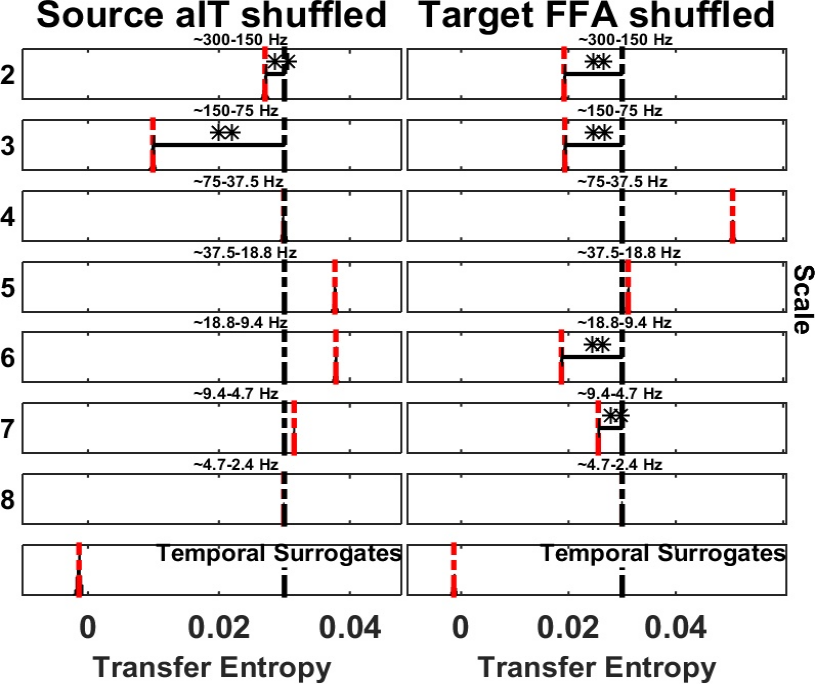

Supplement: S2 Fig — See Fig 4 for display conventions. Spectrally resolved information transfer between aIT as a source and FFA as a target in the condition where subjects are trying to detect target houses. aIT sends information mainly at 75-150Hz (left column), whereas FFA receives information at high frequencies (75-150Hz and above) as well as low frequencies (9-19Hz and 5-9Hz) (right column). (TIFF) [file pcbi.1008526.s002.tiff]

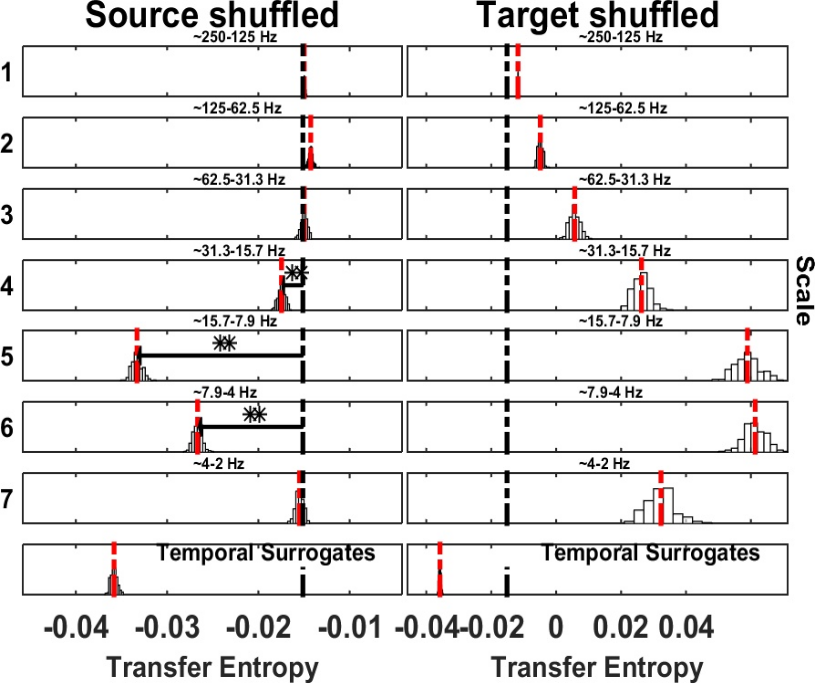

Supplement: S3 Fig — See Fig 4 for display conventions. (Left column) At the source site the maximum drop of the wavelet coefficients is at scale 5. (Right column) The distributions of surrogates at the target site exhibit less increase compared to the ones in Fig 10B, obtained with the block resampling method, especially at lower scales. (TIFF) [file pcbi.1008526.s003.tiff]
