# Supplementary material for: Measuring spectrally-resolved information transfer
Source: PLoS Comput Biol. 2020 Dec 28;16(12):e1008526. doi: 10.1371/journal.pcbi.1008526 (PMC7793276; doi:10.1371/journal.pcbi.1008526)
Supplement: Attachment — Submitted filename: Response_to_reviewers_SpectralTE_PLOSCompBiol.pdf [file pcbi.1008526.s004.pdf]

Point by point replies:

## Reviewer 1: Summary

This study proposes an approach to analysis of cross-frequency information transfer in complex processes constituted by multiple dynamically-interacting elements, particularly neural systems. It is well-argued and exceptionally clearly presented. The relevant literature appears to have been thoroughly researched.

The authors identify the issue that more conventional frequency analyses of information transfer (such as spectral Granger causality) are confined to within-band information transfer, whereas information transfer is potentially many-to-many across the frequency spectrum. The authors correctly recognise that this problem cannot be addressed simply by pre-filtering, and present a solution which uses the maximum overlap discrete wavelet transform (MODWT) to construct surrogate data to instantiate appropriate null distributions for statistical inference. This procedure is validated on synthetic "ground-truth" data, and applied to some neurophysiological datasets, with novel results.

### General points

- While acknowledging that this is common practice in some disciplines, to my mind the language of complex, especially neural, processes, as performing "computation" sits a little uneasily, especially with a neuroscience audience. "Computation" (to me at any rate) suggests an end-to-end algorithm with known input, output and intent. From a neuroscience perspective in particular, the more neutral term "function" (cognitive, behavioural, etc.) feels more appropriate. Not a big deal, though.

**REPLY: We thank Dr. Barnett for pointing out that talking only about distributed computation seems to limit the scope of the proposed methods and may also be misunderstood by some audiences. We have now explicitly added a sentence to the introduction to emphasize that an analysis of information transfer is useful beyond 'distributed computation'.**

- One concern I have is the emphasis on putative partial information decomposition (PID); I say "putative", since, as the authors fully acknowledge, no entirely satisfactory PID has been found to date. More worryingly, it is not even clear that satisfactory PIDs actually exist at all, less so PIDs which, like Shannon (mutual/conditional) information are invariant under transformation of (continuous-state) variables - i.e., how quantities are measured. In my opinion, bringing PID into the picture presents something of a distraction, and potentially a hostage to fortune; this work stands its ground without reference to (possibly chimerical) PIDs.

**REPLY: We agree that the previous version of the manuscript may have suggested to some readers that our method somehow depended on the existence of a PID (for continuous variables). We have now stressed that this is**

**not the case by clarifying this in the corresponding section on PID and in the Introduction section. We hope that it now becomes more clear that PID is useful as a conceptual tool to better interpret the results of a frequency-resolved transfer entropy analysis.**

- One point that should be clarified, is the extent to which “side effects” of the surrogate construction might introduce statistical artefacts. Suppose,e.g., we have a three-variable “indirect” information transfer structure X -> Y -> Z (but not directly X -> Z); i.e., so that T(X -> Z) > 0, but T(X -> Z | Y) = 0. If we wish to discover by the surrogate method whether source frequency-specific T(X -> Z | Y) is significantly different from zero, we would presumably construct (frequency-specific) surrogates for the source X. But in doing so, we also inadvertently impose source frequency T(X -> Y) = 0. Might this confound inference on T(X -> Z | Y)? Similarly for target frequency TE and the “SOSO” procedure. It seems to me that the synthetic examples supplied do not address this situation, or more generally the interaction of surrogate construction with conditioning, which is a necessity for principled analysis of direct information transfer in multivariate scenarios.

**REPLY: We agree that this would be a problem because one would impose a wrong (too strong) null-hypothesis: T(X->Z|Y) = 0 AND T(X->Y)= 0 instead of imposing only the first part. Yet, this problem only arises if one intended to use the spectral methods to also identify the network (i.e. the direct connections). We have now added a subsection to the background stating more clearly that the spectrally-resolved transfer entropy is only to be computed on ‘direct links’ in the network, such that neither cascades X->Y->Z, nor common drivers effects play a role. For network identification proper we now state more clearly that a multivariate transfer entropy analysis must be performed first.**

- Not to be taken as a criticism, but an interesting omission in this study is an analytic, quantitative description of cross-frequency TE; the study presents purely an inferential procedure. Conventional TE (and, e.g., spectral Granger-Geweke causality) have appealingly intuitive quantitative population statistics, which not only may be tested in sample, but stand as “effect size” for information transfer (clearly the authors do appreciate this, when they say “The advantage of the proposed algorithms of avoiding filtering of the original data comes at the price that the measurement of the frequency-specific TE contribution is not in absolute terms, but as a difference to the TE value obtained from the surrogate data”). But does that price need to be paid? Do the authors believe population statistics exist for their cross-frequency TE?

**REPLY: At the presented stage we do not have a formal population statistic for frequency specific transfer entropy. Rather the proposed method is intended to serve as an operational definition of this quantity. Frequency specific TE should be the part of transferred information that vanishes upon scale specific scrambling of wavelet coefficients. This operationalization is corroborated by our analysis of a selection of model system for which the method yields the expected answers. We agree however that the formulation of a population**

**statistic would be a worthwhile addition to the proposed method. We added a paragraph describing the population statistics we're aiming at in a semi-formal way to the section on PID. The main obstacle in defining a proper formal population statistic lies in defining what an isolated frequency component of a random process is exactly.**

- I would like to see some discussion of the impact (if any) of non-stationarity on the method.

**REPLY: Just as in the case of time-domain GC or TE analysis an assumption of stationarity is required because non-stationarity may make the cross-frequency information transfer time-dependent. This in turn considerably complicates the estimation process because collecting realization across time is not valid anymore. Rather access to ensemble data is required. We have included some discussion of this in the manuscript.**

An alternative (although not directly comparable) potential "cross-frequency" approach is via the amplitude (not phase) envelope constructed from the analytic signal of a band-limited signal. This has been, e.g., applied to ECoG data, where there is good evidence that the high-frequency envelope correlates well with average neural firing rates. See e.g., Norman et al., Nature Communications 8(1301), 2017. Perhaps some discussion could be included on this.

**REPLY: We have now promoted the paragraph "A cautionary note on frequency specific information transfer versus cross-frequency coupling" from the discussion section to a higher level and extended the discussion of the relation of other cross-frequency methods to our method. In line with our previous work on this particular topic (Aru et al., Current Opinion in Neurobiology, 2015) we now clarify how cross-frequency methods indeed measure some approximation of mutual information (statistical dependency), instead of (physical) coupling, and can thus be seen as coarse approximations of spectrally-resolved information transfer, except for aspects of directionality, considerations of multivariate structure and measures of information proper (i.e. on the scale of log-probability-ratios).**

**Unfortunately we could not identify the relation between the provided reference of Norman and specifically *cross*-frequency analysis techniques, other than that a frequency specific signal power envelope can be computed and is used in a follow up analysis. We have now added a paragraph to the discussion that discusses the possibility of using power envelopes (which should be necessary when using TE), and hope to have addressed the above suggestion.**

**Specific points**

- p 4, para 2: The authors note that calculation of mTEtot is NP-hard. It may be worth remarking that in practice the situation is even more vexed, since (especially in neural systems) we rarely have access to all potentially-relevant exogenous/latent information sources, that these sources are likely to be far too numerous to account for even in principle, and that measured sources/targets are likely to be spatio-temporal aggregates of "real" (physical) sources.

**REPLY: These are indeed important caveats for real-world systems. We have extended the corresponding section accordingly to alert the reader of these difficulties.**

- p 17, penultimate para: Since (sample) TE cannot be assumed to be normally distributed, is a t-test appropriate for testing mTEtot, or might it be more appropriate to use a nonparametric test (e.g., Mann-Whitney, or some such)?

**REPLY: We apologize for the unclear documentation of the statistical testing. A non-parametric, randomization test was used with t-values serving as the test-statistic, but using a null-distribution of t-values derived via randomization. This is now stated more clearly in the text. In fact, all statistical test performed in our study were non-parametric tests. We have now clearly documented this.**

## Reviewer #2:

This work proposes a new method to identify frequency-specific information transfer between coupled signals, based on comparing transfer entropy (TE) measures computed on two original signals and on surrogate signals obtained destroying the oscillatory structure within specific frequency bands. The method is tested in several simulation settings, and applied to MEG and LFP recordings.

**Main comments:**

- The Authors present as a main advantage of the proposed method the fact that the original signals are unfiltered before computing the information transfer. Indeed, it is known that filtering can have adverse effects on the computation of information transfer and more generally Granger-causal (GC) measures of directed coupling (refs 5-7). However, filtering is applied at the level of surrogate data to rule out some specific temporal scales from the signals prior to computation of the same TE measure applied to the original signals, and inferences about frequency specific directed coupling are made based on the comparison between original and surrogate signals. Therefore, since filtering is actually exploited to make inferences about information transfer, it is not very well clear how the related problems are circumvented in the analysis proposed here.

**REPLY: Dear Prof. Faes, we thank you for highlighting the difficulty for readers to spot the exact reason why filtering problems are 'avoided' in our proposed method. Indeed the fact that we also use a frequency selective technique**

**(MODWT) -akin to filtering- may raise suspicions whether inference on frequency-selective information transfer is not influenced in manner similar to the well-known artifacts. Our answer has two aspects. First, filtering related problems problems are partly avoided as we do not try to diminish the power of the signal(s) to simulate the null hypothesis, but only rely on scrambling phases, i.e. temporal relations. In a nutshell, our null-hypothesis is about temporal order, not signal power. Second, since the phases are only touched in the creation of the surrogate signals we also prevent filtering related problems with respect to the original TE; artifacts arising in the filtering step in our approach will make the method more conservative, i.e. they potentially underestimate the information transfer. We have now stated this more clearly in the introduction section, and also discuss it in a separate subsection of the discussion titled: "Why are the values for spectral mTE sometimes systematically elevated for the surrogate data?".**

- In relation to the comment above, it is known that linear Granger causality is theoretically invariant under filtering, and therefore differences between original and filtered GC measures are due to estimation problem (e.g., ref. 7). Thus, the question arises about what are the mechanisms that allow to reveal "real" (i.e. not due to filtering effects in the application to surrogates) information transfer in this TE application: can they be related to nonlinearities captured by the model free approach, or by the use of the MODWT method for surrogate generation?

**REPLY: As the reviewer correctly suspected it is the use of the MODWT, and the process of 'subtracting' the surrogate TE-values from the true TE-values, that yields the frequency resolution. In other words, as that laid out above the mechanisms to reveal "real" information transfer is via destroying temporal order - but only in a frequency specific way - in the surrogates. We use the wavelet approach basically only to get a frequency specific handle on temporal order. We also point to the discussion on PID, where we state which parts of the total information transfer are revealed by our method (i.e.unique and synergistic information transfer of the frequency of interest) and by a hypothetical, ideal filtering method (unique and redundant information transfer at the frequency of interest). As stated already in our reply to the previous point, our method is not entirely free of distorting effects of the frequency-selective steps; yet, the effects are moved to the surrogate data, where they make the method more conservative.**

- In my understanding, the proposed method allows to detect whether significant information transfer is present between specific time scales in two signals, but does not test the significance of the information transfer against the zero level. To clarify this issue, it would be appropriate to state explicitly the null hypothesis against which the different algorithms are tested, and to report whether and how the evaluation of statistically significant information transfer has been performed in both simulations and real data applications.

**REPLY: The significance of (multivariate) information transfer against a zero-level must indeed be tested prior to applying our approach. For the data presented in our manuscript this was done using the statistical tests and the iterative algorithm presented in Novelli et al., Network Neuroscience, 2019. We have changed the text to reflect the two-stage nature of our approach more clearly by adding a new section "Problem Statement and Analysis Setting" in the Background part of the manuscript.**

**The null hypothesis tested by the approach presented for the information sent by specific source frequencies is: "The wavelet-coefficients of the frequency-component of interest in the source time series are exchangeable in their temporal relation to the target time series with respect to the information transferred between source and target". For the information transfer received by a target frequency the corresponding null-hypothesis is obtained by exchanging 'source' and 'target'.**

- Simulations exhaustively test the frequency-specific information transfer in situations of unidirectional coupling between two signals. However, simulations are not reported about bidirectionally coupled signals, nor regarding multivariate signals where typical confounders (e.g., common drivers, cascades) are introduced. It would be desirable to see how the proposed method behaves in these settings.

**REPLY: Indeed this is a very important point, especially for the analysis of neural data. In fact, there are two aspects to this question: (1) The analysis of the presence of information transfer from source to target in networks with feedback, and (2) the typical confounding factors such as common drivers and cascades in a multivariate setting – this second aspect is handled via the initial analysis of multivariate information transfer (mTE), as detailed in Novelli et al., Network Neuroscience, 2019. An analysis of this kind must precede the application of our novel algorithm. Potential confounders in the set of possible sources are thus already part of the additional conditioning set of sources $\{S \setminus S_i\}$ in the conditional mTE(S_i->Y|S\S_i).**

**Yet, the question indeed remains (and is very important) how our new algorithm performs under more complex network scenarios. We have therefore added a simulation of a bidirectionally coupled system.**

- It would be also good to report more details about the estimation of TE in terms of description of the estimator chosen, setting of its specific parameters, and about the data requirement (appropriate length of the time series which allows reliable TE estimation)

**REPLY: We have now added these important details to the methods section. Specifically, we added details on TE estimation and the number of samples used. Note, that the numbers used do not represent lower bounds on the amount of data needed. We think that for the AR processes as they were used in the simulations the lower bound for detecting frequency-specific TE will be**

**on the order of 5000-10.000 samples (compare to Wollstadt et al., PLOS One, 2014, Fig. 8).**

- In the simulation of coupled Roessler systems, no significant drop was observed for the TE at the target site; however, in Fig. 10 it seems that the TE is significantly below the surrogate distribution. How would you interpret this apparently higher information transfer on the surrogates?

**REPLY: We consider this to be an effect of the well-known filtering problems manifesting on the side of the surrogate data for this system. We now elaborate on this observation in a dedicated subsection in the discussion. In the supplementary material we now also show that this effect can be influenced via the surrogate data construction, and is thus indeed a filtering artifact.**

- In the final part of the paper where the relation to previous approaches is discussed, the Authors may want to discuss also the conceptual differences of their method with the linear and model free measures of multiscale Granger causality and information transfer introduced in [Faes, L., et al. (2017), Physical Review E, 96(4), 042150] and in [Wan, X., & Xu, L. (2018), PloS one, 13(12), e0208423].

**REPLY: Thank you for pointing out these interesting recent developments! We have added them to the discussion section.**

**Technical/minor:**

- The simulation figures are probably too many; a condensed representation of the simulation results (e.g., one figure per simulation) could be envisaged; the same may hold for the second application on real data.

**REPL: Yes, indeed, more condensed figures keep the material closer together - we have changed the figures accordingly.**

- The time t should appear in the cosine terms of eqs. 10-11, 17-18, and others.

**Reply: We thank the reviewer for catching these typographical errors and have corrected them.**

## Reviewer #3:

This work introduces a method for identifying spectral band specific transfer entropy based on the statistical tests with the surrogate data from discrete wavelet transforms. The method involves five steps of computation, relies on several free parameters (e.g. time lag and number of discrete wavelet components), and is

computationally expensive. However, the method is shown to uncover interesting insights into the dynamics of frequency-specific interactions. The validity of the method is demonstrated with some synthetic data generated from coupled systems of sinusoidal oscillators and time-delay coupled nonlinear oscillators. The method is also applied to real data (human MEG and ferret lfps). The main novelty of the method is the combination of the transfer entropy measure and frequency-specific surrogate data testing, which is claimed to avoid filtering of the original time series. The method has not be compared with any other existing techniques even for linear systems. I have the following comments and questions.

1. The method needs to be tested first in an uncoupled system of oscillators to show how the transfer entropy behaves with wavelet data surrogates.

**REPLY: We have simulated the null case 500 times and computed the resulting false positive rate at a critical alpha-level of 5%. This has now been added to the evaluation section.**

2. How does the method work with the data from a system of interacting AR models with single frequency peak at the source and/or target? Are the results comparable to the results from spectral Granger causality method?

**REPLY: We have simulated the corresponding systems and added a comparison to Granger Causality to the Evaluation section. We find that for linear systems with a within-frequency coupling our method behaves like the Granger Causality method - as expected. Also as expected, in a system with coupling between different scales our method detects the associated information transfer, whereas the standard Granger Causality does not.**

3. The wavelet method is used to uncover time-varying spectral features. The proposed work seems to focus on stationary (non-time varying) processes.

**REPLY: Even a stationary process exhibits an evolution of phases of the wavelets coefficients over time and this evolution in our approach is scrambled relative to the target signal. Put differently, we're not interested in scrambling a fluctuating power time series, but aim at scrambling temporal order, i.e. phase, with respect to a reference signal. This is what we need the wavelet formalism for as a technical tool. Nothing in our approach requires the processes in question to be non-stationary.**

4. This work advocates against filtering of original signals. However, there are no or little issues with filtering producing artifacts if appropriately applied to long time series. These results (at least from a simple model of sinusoidal oscillators) need to be compared with the transfer entropy results obtained from filtered data in some of the simple models.

**REPLY: Even if the filtering (applied correctly for long time series) does not produce (phase) artefacts, the second issue with filtering still applies as detailed in the cited Barnett & Seth reference, i.e. reducing power of a spectral component via filtering does not change its information content when treating**

**the problem analytically (using the differential conditional mutual information). When using a digitized version of the signals, effects of 'correct' filtering depend on numerical precision. They may still be too small (not yielding the desired frequency resolution); they may also become too big, possibly introducing unwanted phase shifts in more complex signals.**

I cannot recommend the manuscript in the present form for publication.

Yours sincerely, on behalf of all co-authors,

Prof. Dr. Michael Wibral
