# Supplementary material for: Measuring spectrally-resolved information transfer
Source: PLoS Comput Biol. 2020 Dec 28;16(12):e1008526. doi: 10.1371/journal.pcbi.1008526 (PMC7793276; doi:10.1371/journal.pcbi.1008526)
Supplement: Attachment — Submitted filename: Response_SpectralTE_PLOSCompBiol_minor.pdf [file pcbi.1008526.s005.pdf]

Georg-August-Universität Göttingen

Campus Institut für Dynamik Biologischer Netzwerke (CIDBN)

Georg-August-Universität – Datengetriebene Analyse biol. Netzwerke – Kellnerweg 6 - 37077 Göttingen

Prof. Dr. Michael Wibral

Georg-August-Universität Göttingen
Campus Institut f. Dynamik biol. Netzwerke
Datengetriebene Analyse biologischer Netzwerke
Kellnerweg 6
D - 37077 Göttingen, Germany

T: + 49 (0) 551 / 39 - 26661
E: michael.wibral@uni-goettingen.de

Sekretariat Bianca Spitalieri
T: + 49 (0) 551 / 39 - 26664
F: + 49 (0) 551 / 39 - 26613
E: wibral_lab@uni-goettingen.de

PLOS Computational Biology
Editorial board

Göttingen, 05. October 2020

Manuscript Submission

Dear Prof. Marinazzo, dear Prof. Barnett, dear Prof. Faes, dear reviewers,

we would like to thank the reviewers for again carefully evaluating our manuscript. We have now adressed the remaining minor issues related to the use of two different approaches to Granger causality and to the spectral resolution of the method. Please find below our point by point reply.

Comments to the Authors:

Reviewer #1: I would like to thank the authors for their considered responses to the points raised in my initial review. I am happy to recommend publication,

Reviewer #2: The Authors have addressed thoroughly and satisfactorily all concerns raised. The revised manuscript is a substantial improved version of the original work, that was already novel and of good quality.

Reviewer #3: -The authors make claim that spectral Granger causality is not able to uncover the causal influences in the case of cross-frequency coupling. In their examples, they used the parametric approach to Granger causality for the case of unidirectional coupling (Figure 6), did not use that approach for the case of cross-frequency coupling but instead used the nonparametric approach to show the negative result (Figure 7). There are additional steps (e.g. multi-taper method) needed for the nonparametric approach to create the spectral matrix. Those details are missing. I do not understand why the

same parametric (or nonparametric) approach was not used for both examples to compare.

Reply: In general we prefer to use Granger approaches that do not rely to heavily on specific assumptions. Thus, the use of the non-parametric approach for the cross-frequency example. In this example, indeed the description the Granger approach was incomplete; we have fixed this now. **The AR(2) case is an exception to our general preference, because in that example the model is known exactly. If the reviewer explicitely requests using the same method on both examples we could supply the nonparametric estimates for the AR(2) case.**

- The method cannot necessarily pinpoint a specific frequency in directed connectivity but can identify a frequency range, the extent of which is not discussed in the manuscript. This limitation needs to be discussed in the main text

Reply: **We have now disscussed this point in the paragraph: Methodological advantages and drawbacks of the proposed method**

Yours sincerely, on behalf of all co-authors,

Prof. Dr. Michael Wibral
